# *AgGMP* encoding GDP-D-mannose pyrophosphorylase from celery enhanced the accumulation of ascorbic acid and resistance to drought stress in *Arabidopsis*

**DOI:** 10.7717/peerj.12976

**Published:** 2022-02-24

**Authors:** Yan-Hua Liu, Hao Wang, Jie-Xia Liu, Sheng Shu, Guo-Fei Tan, Meng-Yao Li, Ao-Qi Duan, Hui Liu, Ai-Sheng Xiong

**Affiliations:** 1 State Key Laboratory of Crop Genetics and Germplasm Enhancement, Ministry of Agriculture and Rural Affairs Key Laboratory of Biology and Germplasm Enhancement of Horticultural Crops in East China, College of Horticulture, Nanjing Agricultural University, Nanjing, Jiangsu, China; 2 Suqian Academy of Protected Horticultures, Suqian, China; 3 Institute of Horticulture, Guizhou Academy of Agricultural Sciences, Guiyang, China; 4 College of Horticulture, Sichuan Agricultural University, Chengdu, China

**Keywords:** *Apium graveolens*, *Arabidopsis thaliana*, Ascorbic acid, GDP-D-mannose pyrophosphorylase, Drought stress, Gene expression

## Abstract

Ascorbic acid (AsA) is an important nutrient in celery, the conversion of D-mannose-1-P to GDP-D-mannose catalyzed by GDP-D-mannose pyrophosphorylase (GMPase) represents the first committed step in the biosynthesis of AsA. To clarify the function of the *AgGMP* gene of celery, the *AgGMP* gene was cloned from celery cv. ‘Jinnan Shiqin’ . It contains an open reading frame (ORF) with the length of 1,086 bp, encoding 361 amino acids. AgGMP protein was highly conserved among different plant species. Phylogenetic analysis demonstrated that the GMP proteins from celery and carrot belonged to the same branch. AgGMP protein was mainly composed of three α-helixes and certain random coils. No signal peptide was found in the AgGMP protein. The subcellular localization indicated that the AgGMP protein was located in the cytoplasm. The relative expression levels of *AgGMP* in ‘Jinnan Shiqin’ were significantly up-regulated at 2 h and 4 h under drought stress treatments. AsA contents in transgenic *Arabidopsis* lines hosting *AgGMP* gene were higher than that in wild type plants, and the root lengths were also longer in the MS medium containing 300 mM mannitol. The present study provides useful evidence for the functional involvement of *AgGMP* in regulating AsA accumulation and response to drought stress in celery.

## Introduction

GDP-D-mannose pyrophosphorylase (GMPase) is a rate-limiting enzyme in the L-galactose pathway, a key biosynthetic pathway for L-ascorbic acid (AsA) in plants. In this pathway, the initial step is the formation of GDP-D-mannose, which is catalyzed by GMPase (Wang et al., 2011). AsA is a water-soluble antioxygenic organic micromolecule widely found in plants. It’s essential for cardiovascular function, immune cell development, connective tissue health, and iron utilization. As a key enzyme in AsA biosynthesis, GMPase in higher plants plays important roles in removing reactive oxygen species (ROS) generated by adverse environmental conditions (Xue et al., 2018), such as high temperature, low temperature (Li et al., 2018a; Liu et al., 2019; Huang et al., 2016), and salt stress (Zhang et al., 2012). In addition, the resistance of abiotic stress in plants could be improved through modulating *GMP* gene expression to increase the AsA content. Until now, *GMP* genes have been isolated from a number of higher plants, including *Arabidopsis thaliana* (Wang, Guo & Tang, 2011), *Camellia sinensis* (Xiao et al., 2015), *Solanum esculentum* (Wang et al., 2011), and *Dendrobium huoshanense* (He, Yu & Tei, 2017).

Plant resistance against abiotic stress was associated with the changes of antioxidant defense system, which consisted of enzymatic and non-enzymatic antioxidants (Garg, Varshney & Jain, 2014; Hasanuzzaman et al., 2017; Li et al., 2018a; Liu et al., 2021). AsA belongs to non-enzymatic antioxidants (Liu et al., 2019), drought stress affects AsA content by controlling the activities of enzymes involved in AsA metabolism (*e.g.* GMP, GalDH, APX, DHAR, MDHAR) (Huang et al., 2016; Liu et al., 2019). AsA can directly remove ROS (reactive oxygen species) produced by stress, and also indirectly remove H_2_O_2_ through the AsA-GSH cycle to protect tissues from harmful oxidative products and to keep certain enzymes in their required reduced forms (Liebler, Kling & Reed, 1986; Padh, 1990; Barth, Gouzd & Steele, 2009), as well as improve the ability of plants to resist abiotic stress.

Under low and high temperature stresses, the relative expression of *SlGMP*, GMPase activity, contents of AsA and dehydroascorbic acid (DHA) were increased, and the content of malondialdehyde (MDA) was decreased in transgenic tomato plants (Wang et al., 2011). The overexpression of *SlGMP* also delayed the senescence of potato (Lin et al., 2011). *OsGMP* gene affected AsA synthesis and GMPase activity in rice (*Oryza sativa*), and overexpression of *OsGMP* reduced the inhibitory effect of NH_4_^+^ on root growth in *A. thaliana* (Li et al., 2010). The *OsGMP* gene also was reported to play a key role in the rice during its nutritional and reproductive stages under salt stress (Kempinski, Haffar & Carina, 2011; Qin et al., 2016a, 2016b). The *GmGMP1* gene can be induced to express under high temperature, low temperature, drought and salt stresses. The overexpression of *GmGMP1* gene in *A. thaliana* and soybean (*Glycine Max*) increases GMPase activity and AsA content, and enhances the ability for eliminating ROS (Xue et al., 2018). The tobacco (*Nicotiana Tabacum*) harboring *PpGMP* showed stronger resistance to salt or drought stresses by increasing the AsA content (Ai, Liao & Li, 2016). *AtGMP* positively regulates the synthesis of AsA. AsA content in transgenic lettuce (*Lactuca sativa*) overexpressing *AtGMP* gene increased to 2.5-fold of that in control (Wang, Guo & Tang, 2011).

Celery (*Apium graveolens*) growth is influenced by multiple environmental factors. In previous studies, it is reported that *GMP* gene participated in plant response to stress. We speculated that *AgGMP* may be involved in celery resisting abiotic stress through modulating the AsA accumulation. However, the characteristics and transcription regulation mechanism of *AgGMP* under drought stress in celery remain unclear. In this study, *AgGMP*, a gene encoding GDP-D-mannose pyrophosphorylase, was cloned from celery, and then its expression patterns were detected in celery under abiotic stress treatments. The transgenic *A. thaliana* plants overexpressed *AgGMP* were obtained to examine the AsA level and compared their root growth with wild-type under drought stress. This study further clarified the roles of *AgGMP* in abiotic stress, and provided a theoretical basis for stress response of celery.

## Materials and Methods

### Plant materials, growth conditions and stress treatments

Celery cv. ‘Jinnan Shiqin’, *A. thaliana* ecotype Columbia (WT), and transgenic *A. thaliana* were grown in pots within a soil/vermiculite mixture in phytotron at the Key Laboratory of Crop Genetics and Germplasm Enhancement, Nanjing Agricultural University (32°03′N, 118°84′E). The phytotron program was 25 °C/18 °C (day/night) for 16 h/8 h. The light intensity was 300 μmol m^−2^ s^−1^ at daytime, with relative humidity of 75%. Two-month-old (day after germinating) ‘Jinnan Shiqin’ plants were grouped and treated with 4 °C, 38 °C, 200 g·L^−1^ PEG 6000 and 200 mM NaCl, respectively. The leaf blades of celery with the longest petiole were collected at 0, 1, 2, 4, 8, and 24 h after treatments. All the samples were frozen in liquid nitrogen immediately and then stored at −80 °C for RNA extraction. WT and *AgGMP*-OE lines were grown on Murashige and Skoog (MS) medium with or without 300 mM mannitol (control). Each experiment was performed with three biological replicates.

### Total RNA extraction, cDNA synthesis and AsA content determination

Total RNA of celery and *A. thaliana* were extracted using the total RNA extraction Kit (Tiangen, Beijing, China) according to the manufacturer’s instructions. cDNA was obtained by Prime Script RT reagent Kit (TaKaRa, Dalian, China) based on the operation instruction. The content of AsA was determined using UPLC (ultra performance liquid chromatography) system according to the method described as previous study (Liu et al., 2019).

### Bioinformatics analysis

The sequences of GMP proteins from other species were downloaded from the National Center for Biotechnology Information (NCBI) database (https://www.ncbi.nlm.nih.gov/). Primer Premier 6.0 was utilized to design primers. Nucleotide and encoded amino acid sequence of *AgGMP* gene was analyzed using BioXM software. MEGA7.0 software was used to construct the phylogenetic tree. The alignment of amino acid sequences of GMPs from celery and other plants were carried out by DNAMAN 8.0 software. SOPMA software (http://pbil.ibcp.fr/) was used to predict the secondary structure of GMP protein. The protein tertiary structural model was established using CPH models 3.2 Server (http://www.cbs.dtu.dk/services/CPHmodels/). Signal peptide was predicted by Signal P software.

### Isolation of the *AgGMP* gene, overexpression vector construction and *A. thaliana* transformation

The putative *AgGMP* gene sequence was retrieved from celery genome and transcriptome database (Feng et al., 2018a; Li et al., 2020c). The full lengths ORF (open reading frame) of *AgGMP* was amplified with special primers (*AgGMP*-7736-F: 5′-TTTACAATTACCATGGGATCCATGAAGGCTCTTATTCTTGTTGGA-3′; *AgGMP*-7736-R: 5′-ACCGATGATACGAACGAGCTCTCACATCACAATCTCTGGCTTCAA-3′). The PCR product was cloned into the pCAMBIA1301 and then sequenced (Genscript, Nanjing, China). The recombinant plasmid (*35S: AgGMP*) was introduced into the *Agrobacterium tumefaciens* strain GV3101 *via* electroporation method. The floral-dip method was used for *Agrobacterium*-mediated transformation of *A. thaliana* (Zhang, Henriques & Lin, 2006). Transgenic *A. thaliana* were initially screened on MS medium containing hygromycin (40 mg/L), and then further confirmed by β-glucuronidase (GUS) assay, PCR amplification and sequencing.

### Subcellular localization

The ORF of *AgGMP* without stop codon was amplified using specific primers (*AgGMP*-PA7-F: 5′-CACCATCACCATCACGCCATGATGAAGGCTCTTATTCTT GTTGGA-3′ and *AgGMP*-PA7-R: 5′-CACTAGTACGTCGACCATGGCCATCACAATCTCTGGCTTCAA-3′). The amplification product was ligated into pA7 vector *via Nco* I site. The control vector (pA7-GFP) and recombinant vector (AgGMP-GFP) were bombarded into onion epidermal cells using the biolistic bombardment Biolistic PDS-1000 (Bio-Rad, Hercules, CA, USA). After 18 h of dark growth on MS solid medium, the GFP fluorescence of samples were observed using a laser confocal microscope LSM780 (Zeiss, Oberkochen, Germany).

### Real-time quantitative PCR analysis

Real-time quantitative PCR (RT-qPCR) was conducted to detect the expression level of *AgGMP*. Premier 6.0 software was used to designed primers (*AgGMP*-qF: 5′-TGCTGGAATCTACCTGCTGAACC-3′, *AgGMP*-qR: 5′-TGCTGGAATCTACCTGCTGAACC-3′). The SYBR Premix *Ex Taq* (TaKaRa, Dalian, China) and Bio-Rad IQ5 real-time PCR System (Bio-Rad, Hercules, CA, USA) were used for RT-qPCR reaction. *AgActin* gene was used as internal standard (Li et al., 2016). Each reaction set three biological replicates. The relative expression data of *AgGMP* were analyzed using 2^−ΔΔCt^ method (Pfaffl, 2001).

### Statistical analysis

All data in the text were obtained from the average of three biological repeats. Data significant difference was analyzed using SPSS 24.0 by one way ANOVA at a 0.05 level.

## Results

### Analysis of *AgGMP* sequence

**Figure 1:**
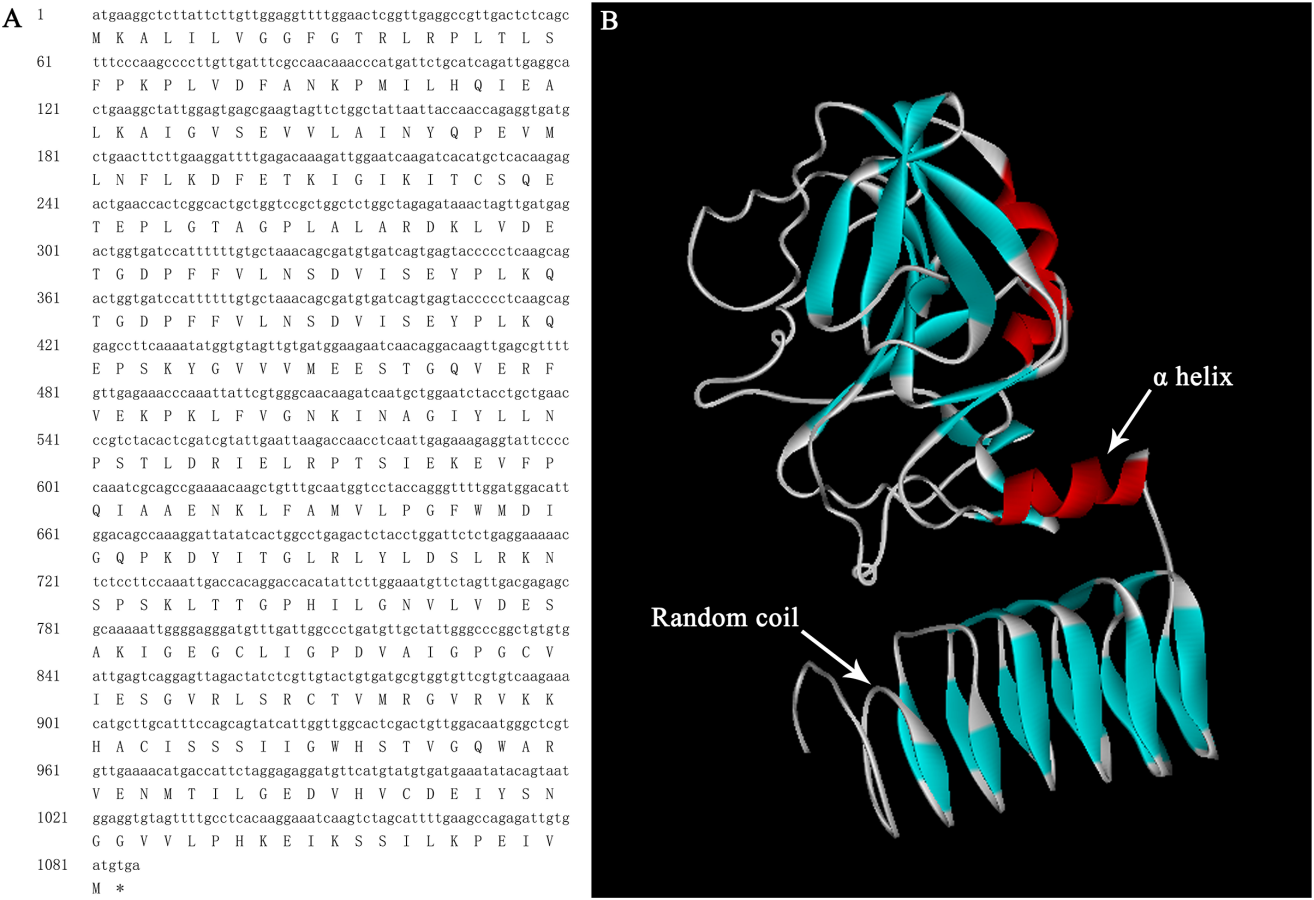
Bioinformatics analysis of AgGMP. (A) Nucleotide and encoded amino acid sequence of *AgGMP* gene. (B) The tertiary structural model of AgGMP protein.

**Figure 2:**
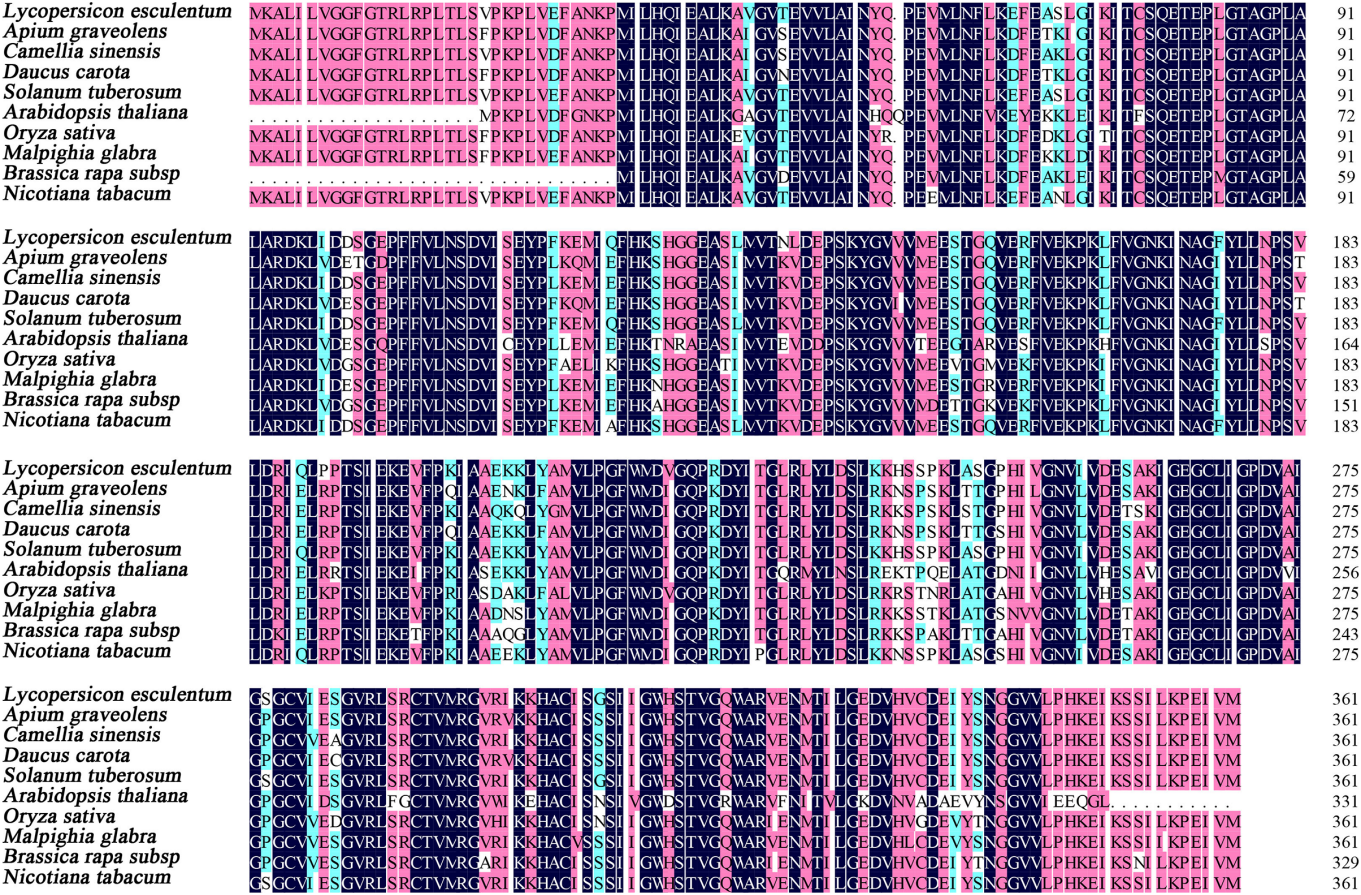
The alignment of amino acid sequences of GMPs from celery and other plant species.

**Figure 3:**
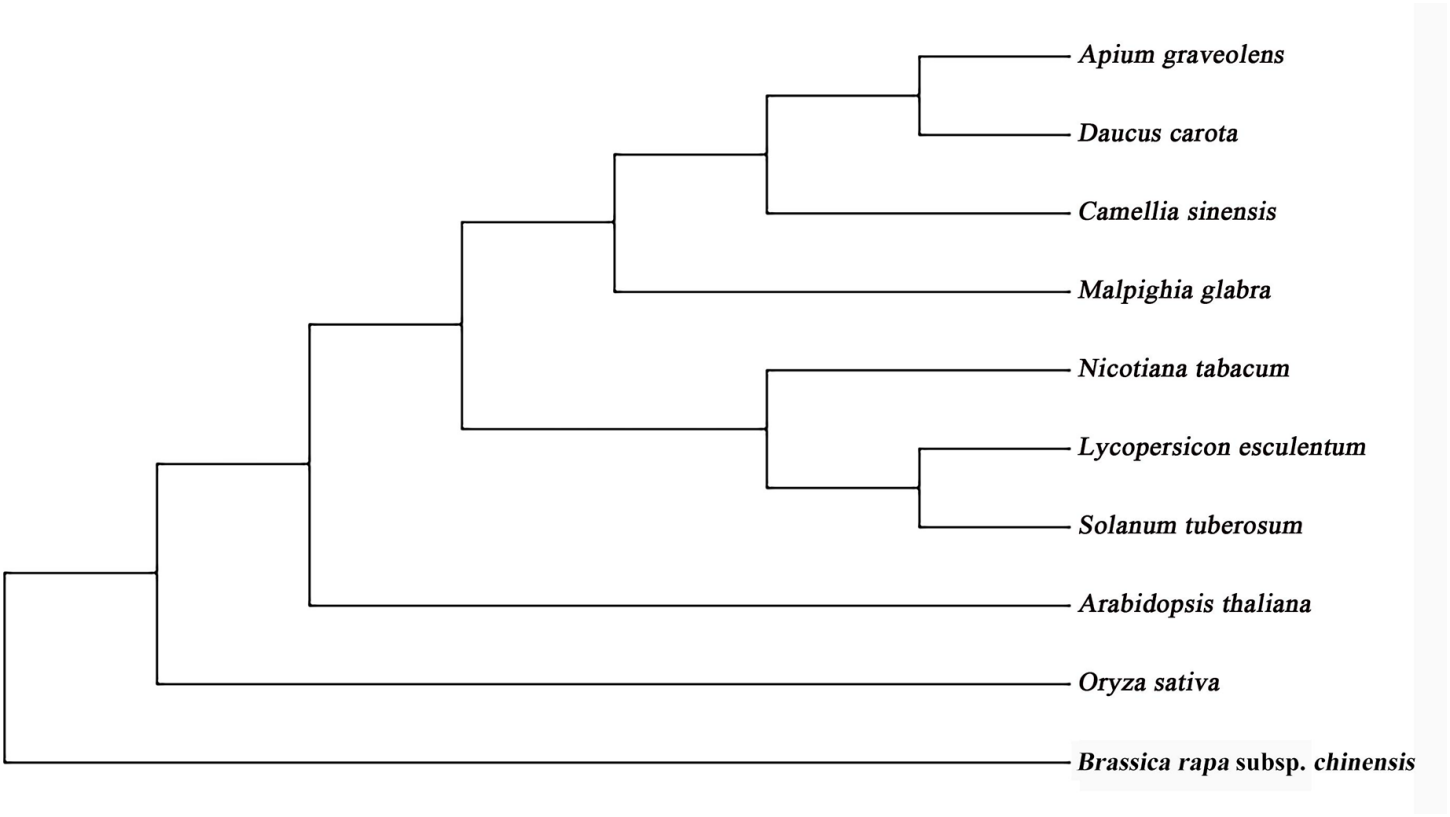
Phylogenetic tree of GMP proteins from celery and other plant species.

The full lengths cDNA sequence of *AgGMP* gene was obtained from ‘Jinnan Shiqin’ (GenBank No. OL757646), which contained an open reading frame (ORF) with the length of 1,086 bp, encoding 361 amino acids (Fig. 1A). The secondary structure was mainly composed of 30.77% α-helixes, 29.09% extended strand, 9.07% β-turn and 37.67% random coils. Prediction results indicated that the protein tertiary structure of AgGMP protein was mainly composed of three α-helixes and certain random coils (Fig. 1B). AgGMP protein has no signal peptide according to the prediction by Signal P software. The amino acid sequence of AgGMP were aligned with homologous sequences from *Daucus carota* (carrot, AQM57027.1), *Lycopersicon esculentum* (tomato, accession ID DQ449030), *Solanum tuberosum* (potato, NP_001275205.1), *Camellia sinensis* (tea plant, AGI78460.1), *Arabidopsis thaliana* (AT4G30570), *Oryza sativa* (rice, LOC4327472), *Malpighia glabra* (acerola, ABB53473.1), *Brassica rapa* subsp. *chinensis* (non-heading Chinese cabbage, AET14212.1), *Nicotiana tabacum* (tobacco, BAB62108.1) (Fig. 2). GMP proteins were highly conserved (90.80% of consistency) among different species. Phylogenetic analysis demonstrated that AgGMP protein had the closest evolutionary relationship with carrot (Fig. 3).

### Expression profiles of *AgGMP* under abiotic stress in celery

**Figure 4:**
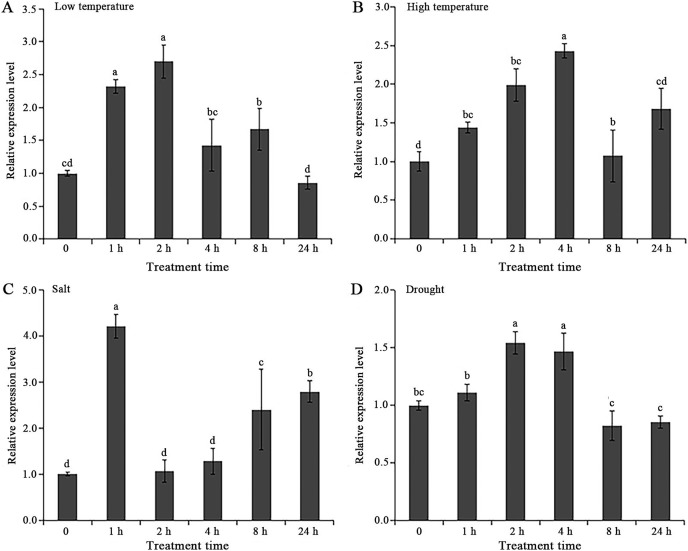
Expression analysis of *AgGMP* in celery under abiotic stress treatments. (A) Low temperature. (B) High temperature. (C) Salt. (D) Drought. Data are expressed as the means ± standard deviation (SD) of three replicates. Different letters indicate significant difference at 0.05 level.

As shown in Fig. 4, the relative expression levels of *AgGMP* gene in ‘Jinnan Shiqin’ were up-regulated significantly under low temperature, high temperature, salt and drought treatments. The relative expression levels of *AgGMP* gene were obviously increased at 1 h after low temperature, high temperature and salt treatments, respectively (Figs. 4A–4C). In salt treatment, the relative expression levels of *AgGMP* gene were remarkably elevated at 8 h and 24 h than that at 0 h, which were 2.52 and 2.79-folds of that at 0 h, respectively. The highest expression of *AgGMP* gene were occurred at 2 h, 4 h, 1 h and 2 h of low temperature, high temperature, salt and drought treatments, which were 2.70, 2.43, 1.54, and 4.2-folds of the 0 h, respectively. Under drought stress, the expression profiles of *AgGMP* exhibited an increase at 1 h, 2 h, 4 h, which were 1.11, 1.54 and 1.47, and then decreased at 8 h, 24 h, which were 0.82 and 0.85, respectively (Fig. 4D). The results indicated that *AgGMP* involved in the response to abiotic stress in celery.

### Subcellular localization analysis of AgGMP

**Figure 5:**
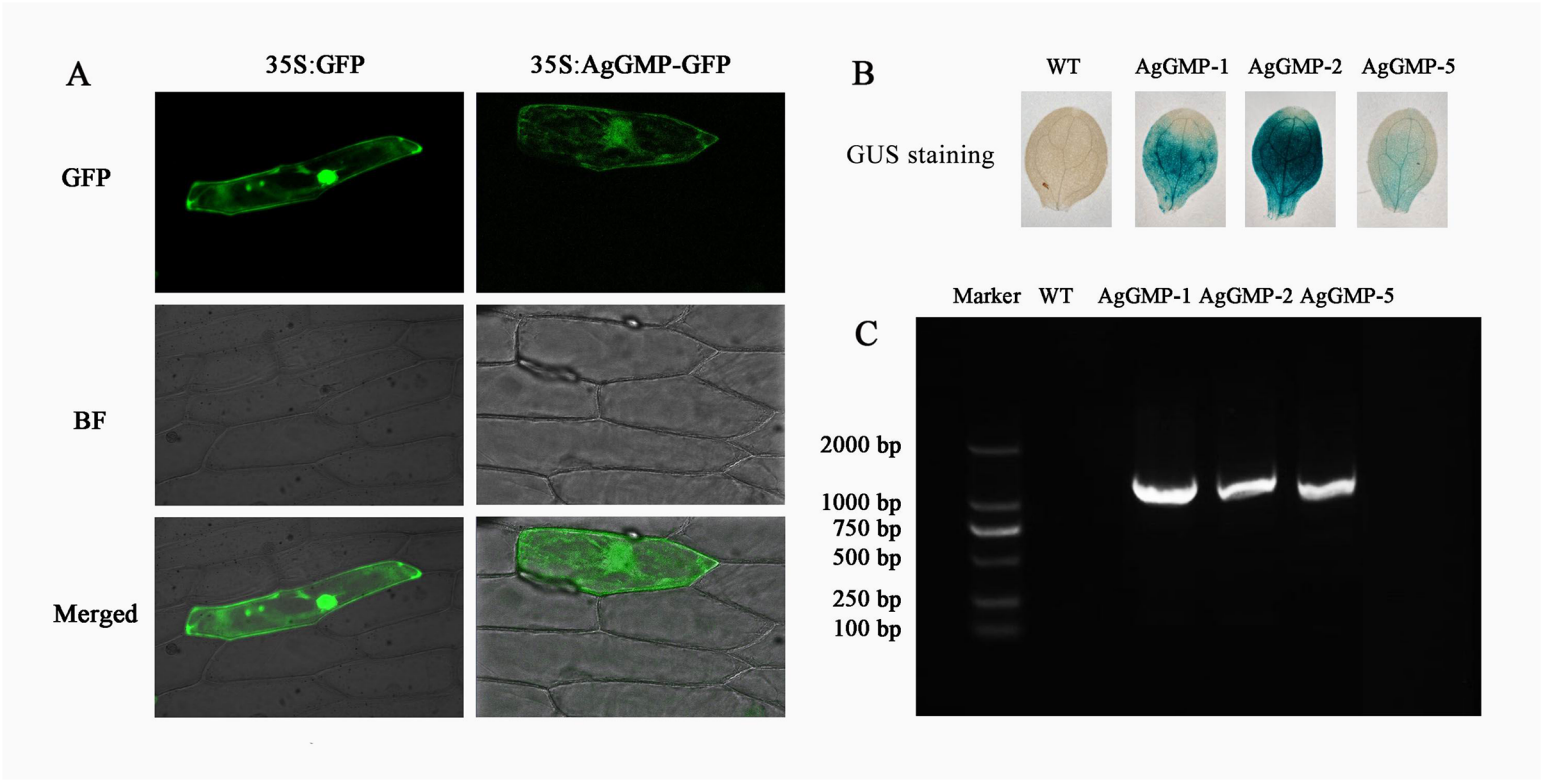
Subcellular localization of AgGMP protein and identification of transgenic *A*. *thaliana* lines. (A) Subcellular localization of AgGMP protein. (B) GUS staining of leaves from transgenic *A. thaliana* and WT. (C) Identification of transgenic *A. thaliana* using PCR amplification.

The vector AgGMP-GFP and pA7-GFP were transferred into the onion epidermis cell to detect the subcellular localization of AgGMP. The onion epidermal cell with AgGMP-GFP and pA7-GFP displayed bright fluorescence throughout the entire cell, suggesting that the AgGMP protein was located in cytoplasm (Fig. 5A).

### Identification of transgenic *A. thaliana*

To investigate the function of *AgGMP* gene, transgenic *A. thaliana* lines were generated *via Agrobacterium*-mediated transformation. The transgenic *A. thaliana* lines (AgGMP-1, AgGMP-2, and AgGMP-5) were screened on the MS medium containing hygromycin. The leaves of transgenic *A. thaliana* lines were immersed in X-gluc (5-bromo-4-chloro-3-indolyl-β-D-glucuronic acid) and appeared blue (Fig. 5B). About 1,000 bp PCR products were observed only in the transgenic lines, AgGMP-1, AgGMP-2 and AgGMP-5, based on the PCR amplification (Fig. 5C). The results indicated that *AgGMP* was successfully transferred into *A. thaliana*, three OE lines (AgGMP-1, AgGMP-2 and AgGMP-5) harboring *AgGMP* gene were obtained.

### Overexpression of *AgGMP* up-regulated the AsA content *in Arabidopsis*

**Figure 6:**
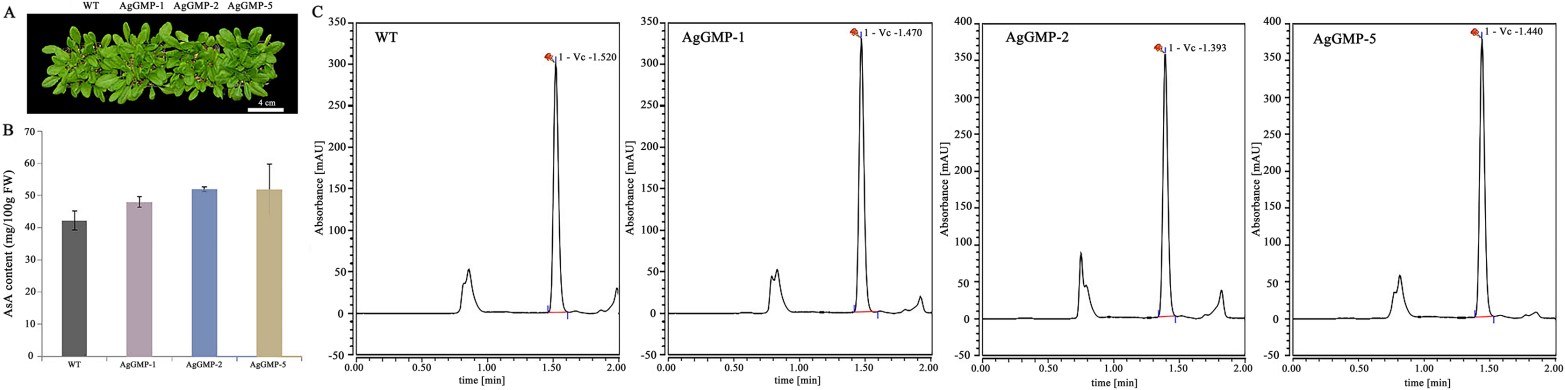
AsA contents of WT and transgenic *A. thaliana*. (A) Phenotype of WT and transgenic *A. thaliana*. Scale bars = 3.5 cm. (B) AsA content. (C) UPLC chromatogram. Data are expressed as the means ± standard deviation (SD) of three replicates.

There are no obvious differences observed in phenotype among three OE lines and WT plants (Fig. 6A). According to the AsA content determination, the transgenic *A. thaliana* lines contained higher AsA accumulation compared with WT (Figs. 6B and 6C). The AsA contents in WT, AgGMP-1, AgGMP-2 and AgGMP-5 lines were 42.25, 48.04, 51.98, and 52.73 mg/100g FW (fresh weight), respectively. The results indicated that overexpression of *AgGMP* up-regulated the AsA level in transgenic *A. thaliana*.

### The change of seedling root lengths in *A. thaliana* under 300 mM mannitol treatment

**Figure 7:**
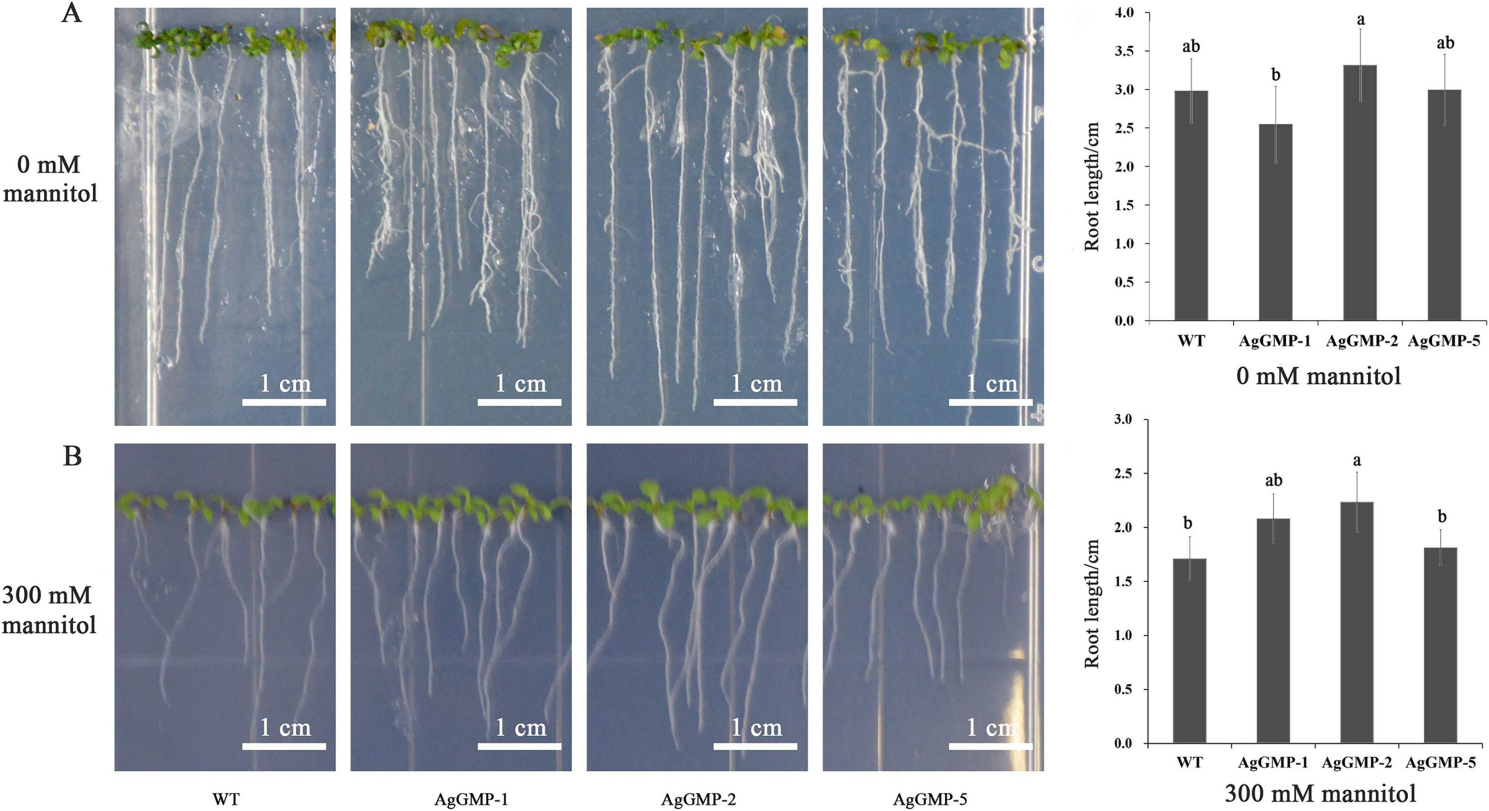
Root growth of WT and transgenic *A. thaliana* lines subjected to mannitol application. (A) Root lengths of transgenic *A. thaliana* and WT on MS medium without mannitol application. Scale bars = 1.5 cm. (B) Root lengths of transgenic *A. thaliana* and WT on MS medium with 300 mM mannitol application. Data are expressed as the means ± standard deviation (SD) of three replicates. Different letters indicate significant difference at 0.05 level.

The seeds of three *A. thaliana* OE lines and WT were germinated and grown on MS medium containing different mannitol concentrations. The root lengths of 7-day-old (day after germinating) *A. thaliana* plants were measured. On MS medium without mannitol, no significant difference in root lengths was observed between WT and transgenic *A. thaliana* lines hosting *AgGMP* gene. The root lengths of WT, AgGMP-1, AgGMP-2, and AgGMP-5 lines, were 2.98, 2.55, 3.31 and 3.00 cm, respectively (Fig. 7A). Whereas, the roots lengths were inhibited in WT and three transgenic *A. thaliana* lines treated with 300 mM mannitol, which were 1.71, 2.08, 2.23, and 1.81 cm, respectively (Fig. 7B). On MS medium without mannitol, the root lengths of *A. thaliana* were longer, more lateral roots and root hairs, as well as better root growth. The root lengths of transgenic plants were longer than that of WT on MS medium containing 300 mM mannitol. In particular, the root lengths of AgGMP-2 were significantly increased compared with WT.

## Discussion

Celery is one of important leafy vegetables with rich nutrients (Kooti & Daraei, 2017; Li et al., 2018b), such as anthocyanin (Feng et al., 2018b, 2021), apigenin (Tan et al., 2017; Yan et al., 2019; Wang et al., 2021), carotenoids (Li et al., 2019; Yin et al., 2020; Ding et al., 2021), AsA (Liu et al., 2021), and dietary fiber (Duan et al., 2020). AsA is not only is one of important nutrients, but also a key mediator that triggers plant response to various abiotic stress (Barth, Gouzd & Steele, 2009). AsA accumulation is affected by abiotic stress, such as salt (Zhang et al., 2012), low temperature, high temperature (Wang et al., 2011), drought stress (Padh, 1990), light (Li et al., 2020a), and a variety of nutrient elements (Li et al., 2020b), which is speculated to accomplished by increasing the activity of related enzymes involved in the AsA biosynthesis pathway in plants.

In plants, there are four pathways for AsA biosynthesis, including L-galactose pathway, *myo*-inositol pathway, L-gulose pathway, and D-galacturonate pathway. The *GMP* gene, encoding GMPase, plays an essential role in the L-galactose pathway. GMPase catalyzes the initial steps of AsA biosynthesis to form GDP-D-mannose, the precursor of AsA. Thus, *GMP* gene could affect AsA content by controlling the activities of GMPase. Our present result indicated that *AgGMP* involved in the response to abiotic stress in celery. The identified function of *AgGMP* is similar to that of soybean *GmGMP1*, *GMP* genes expression were both significantly induced by heat, cold and salt stresses. Under abiotic stress treatments, the expression level of *GmGMP1* peak at 1 h and then decreased (Xue et al., 2018). Under drought stress, the expression levels of the *AgAPX1* and *AgGMP* of celery were also similar, which significantly higher than that of the control at 2 h, and peaked at 4 h followed by a decrease (Liu et al., 2019).

It is acknowledged that drought is an important factor affecting agricultural production, improving plant drought resistance also is an ongoing hot topic. Considering evidences have suggested the relationship between AsA and stress resistance (Wang et al., 2011; Zhang et al., 2012). One of the consequences of abiotic stress is that it triggers an oxidative burst due to formation of reactive oxygen species. AsA could directly remove ROS produced by stress, and also indirectly remove H_2_O_2_ through the AsA-GSH cycle to protect tissues from harmful oxidative products, as well as keep certain enzymes in their required reduced forms. GMPase is a key rate limiting enzymes in AsA biosynthesis. Declined expression of *GMP* gene, encoded GMPase, usually was followed by a decreased resistance to drought stress in plants. Overexpression of the *GMP* gene of soybean enhanced the plant drought resistance in transgenic plants (Xue et al., 2018). In this study, we found that the relative expression levels of *AgGMP* gene were up-regulated significantly under low temperature, high temperature, salt and drought treatments, which reached the peak at 2 h, 4 h, 1 h, and 2 h, respectively. The transcript of *AgGMP* in celery were induced by drought stress, the expression of *AgGMP* in celery was significantly up-regulated of *AgGMP* under drought stress at 2 h and reach peak at 4 h and then declined.

To further investigate the function of *AgGMP* gene in response to drought stress, the AsA content and root lengths of three *A. thaliana* OE lines and WT were measured. Compare with the WT, overexpression of *AgGMP* up-regulated the AsA content in three transgenic *A. thaliana* lines, the root lengths were also longer when subjected to 300 mM mannitol. It is possible that overexpression of *AgGMP* can improve the activity of GMPase that involved in AsA biosynthesis pathway in *A. thaliana*, and increase the AsA accumulation to neutralize part of the stress effects. AsA could remove ROS and H_2_O_2_ produced by drought stress, protect tissues from harmful oxidative products and keep certain enzymes in their required reduced forms (Wang et al., 2011; Zhang et al., 2012). The three transgenic *A. thaliana* lines showed reduced root damage caused by mannitol and presented greater tolerance to drought stress. We speculated that *AgGMP* was participated in the process of celery resisting drought stress and overexpression of *AgGMP* can induce an increased resistance to drought stress in transgenic plants by enhancing the accumulation of AsA.

## Conclusion

GMPase was a rate-limiting enzyme in the L-galactose pathway, a key biosynthetic pathway for L-ascorbic acid (AsA) in plants. Here, the gene *AgGMP* encoding the AgGMPase was identified and characterized. The *AgGMP* gene contained an ORF of 1,086 bp, encoding 361 amino acids. Sequence alignment suggested that AgGMP protein was highly conserved among different plant species. The transcript of *AgGMP* was induced by abiotic stress in celery, the heterologous overexpression of *AgGMP* in *A. thaliana* proved the role of *AgGMP* in regulating AsA accumulation and modifying drought stress resistance. These findings suggested that AgGMP acted a regulator in AsA accumulation and response to abiotic stress in celery. In the future, we hope to achieve its homologous over-expression through transgenic technology and knock it out using the CRISPR/Cas9 method in celery.

## Supplemental Information

10.7717/peerj.12976/supp-1 Supplemental Information 1 Raw data.

## ABBREVIATIONS

AsA: L-ascorbic acid
DHA: Dehydroascorbic acid
GFP: Green fluorescent protein
GMPase: GDP-D-mannose pyrophosphorylase
GUS: β-glucuronidase
MDA: Malondialdehyde
MS medium: Murashige and Skoog medium
ORF: Open reading frame
PCR: Polymerase Chain Reaction
ROS: Reactive oxygen species
RT-qPCR: Real-time quantitative PCR
UPLC: Ultra Performance Liquid Chromatography
X-gluc: 5-bromo-4-chloro-3-indolyl-β-D-glucuronic acid

## Additional Information and Declarations

### Data Availability

The following information was supplied regarding data availability: The raw data are available in the Supplemental File.
